# Supplementary material for: Comparison of Rhizosphere Bacterial Communities of Pinus squamata, a Plant Species with Extremely Small Populations (PSESP) in Different Conservation Sites
Source: Microorganisms. 2024 Mar 22;12(4):638. doi: 10.3390/microorganisms12040638 (PMC11051972; doi:10.3390/microorganisms12040638)
Supplement: Supplementary file 1 [file microorganisms-12-00638-s001.zip › A descriptive caption for each supplementary material.pdf]

A descriptive caption for each supplementary material

Supplementary Table S1 Sample sequencing information

Supplementary Table S2 Relationships at phylum level

Supplementary Table S3 Relationships at genus level

Supplementary Figure S1 Rarefaction curves

Supplementary Figure S2 Linear discriminant analysis

Supplementary Figure S3 COG function classification

Supplementary Figure S4 Function prediction heatmap

Supplementary Figure S5 Functional difference analysis
