# Supplementary figures and images for: Comparison of Rhizosphere Bacterial Communities of Pinus squamata, a Plant Species with Extremely Small Populations (PSESP) in Different Conservation Sites
Source: Microorganisms. 2024 Mar 22;12(4):638. doi: 10.3390/microorganisms12040638 (PMC11051972; doi:10.3390/microorganisms12040638)

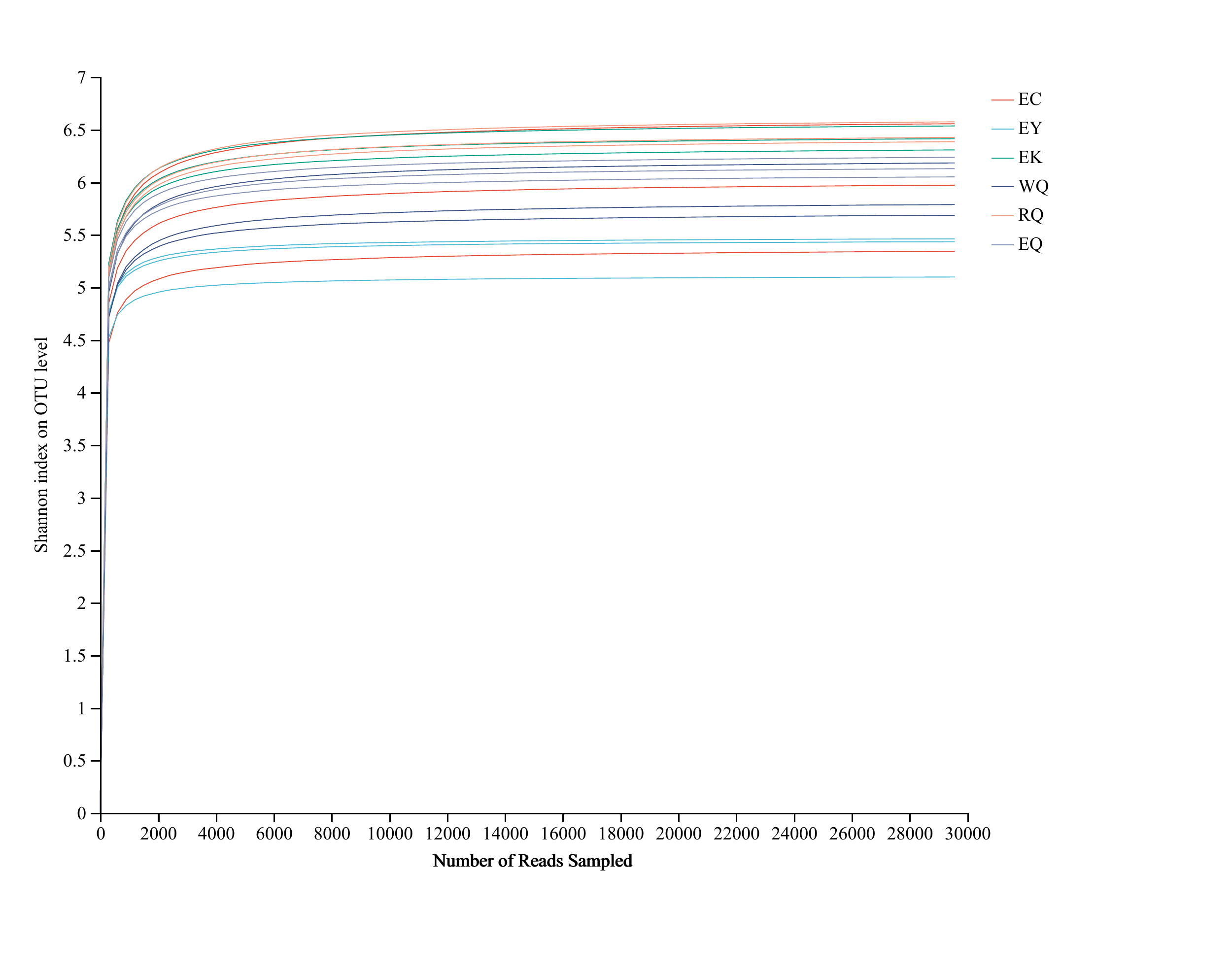

Supplement: Supplementary file 1 [file microorganisms-12-00638-s001.zip › Supplementary Figure S1.tiff]

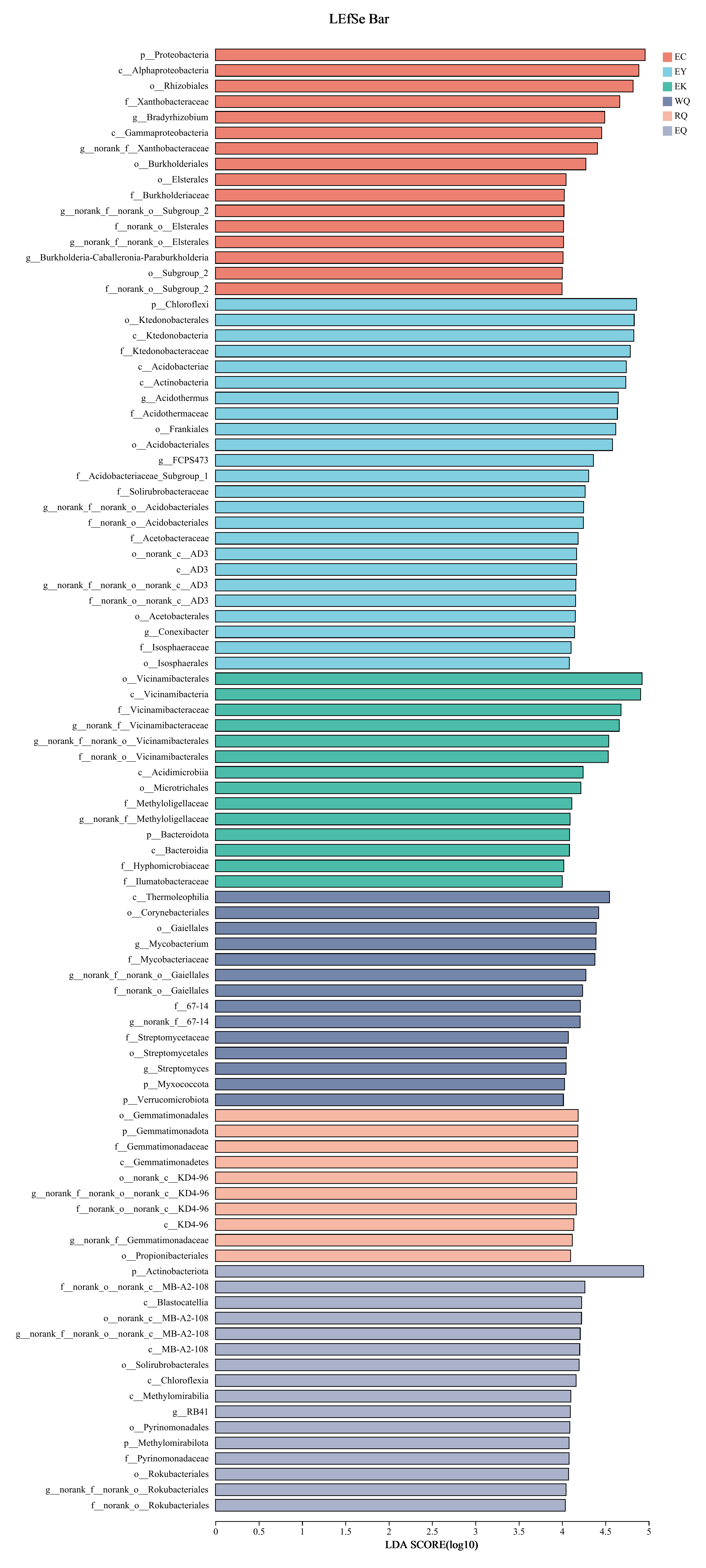

Supplement: Supplementary file 1 [file microorganisms-12-00638-s001.zip › Supplementary Figure S2.tiff]

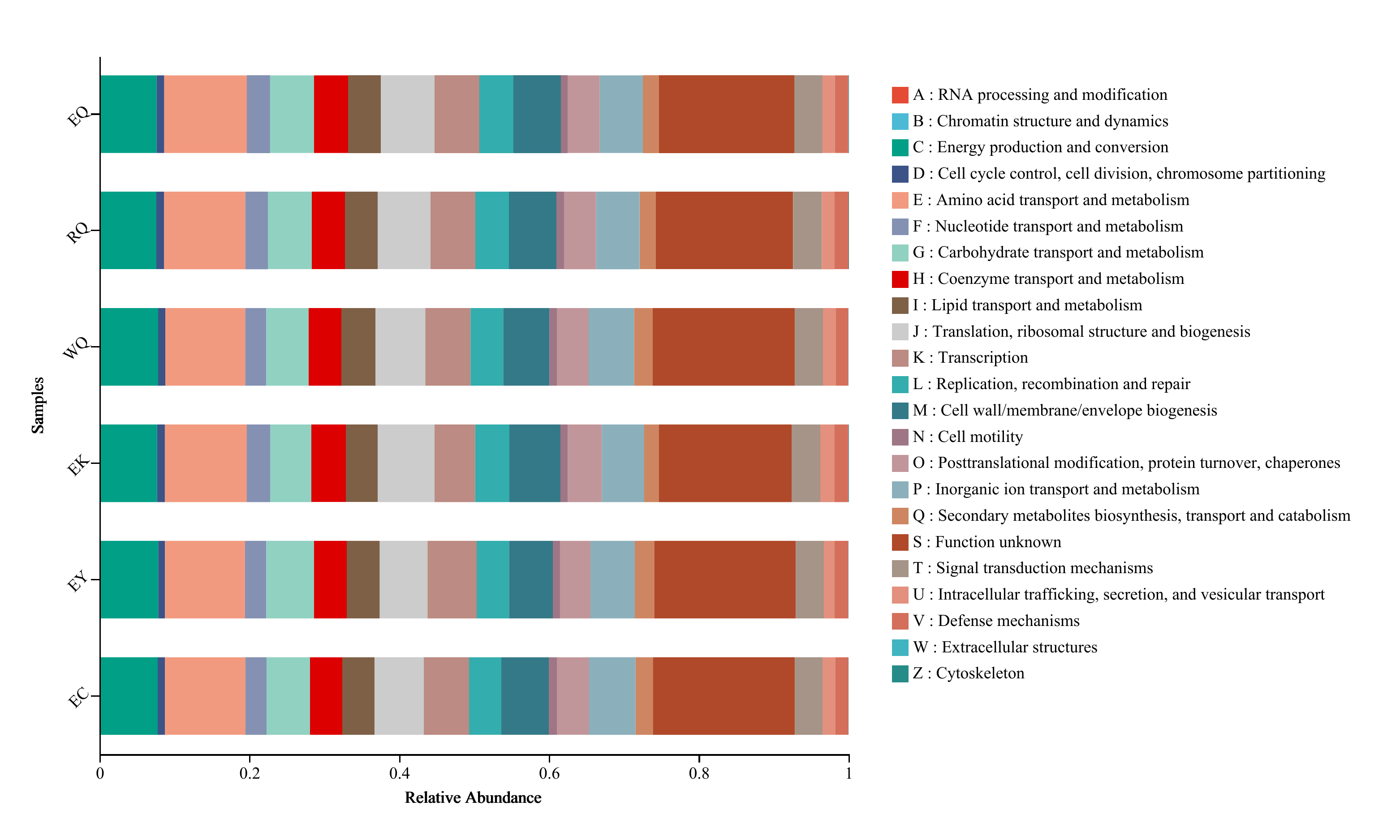

Supplement: Supplementary file 1 [file microorganisms-12-00638-s001.zip › Supplementary Figure S3.tiff]

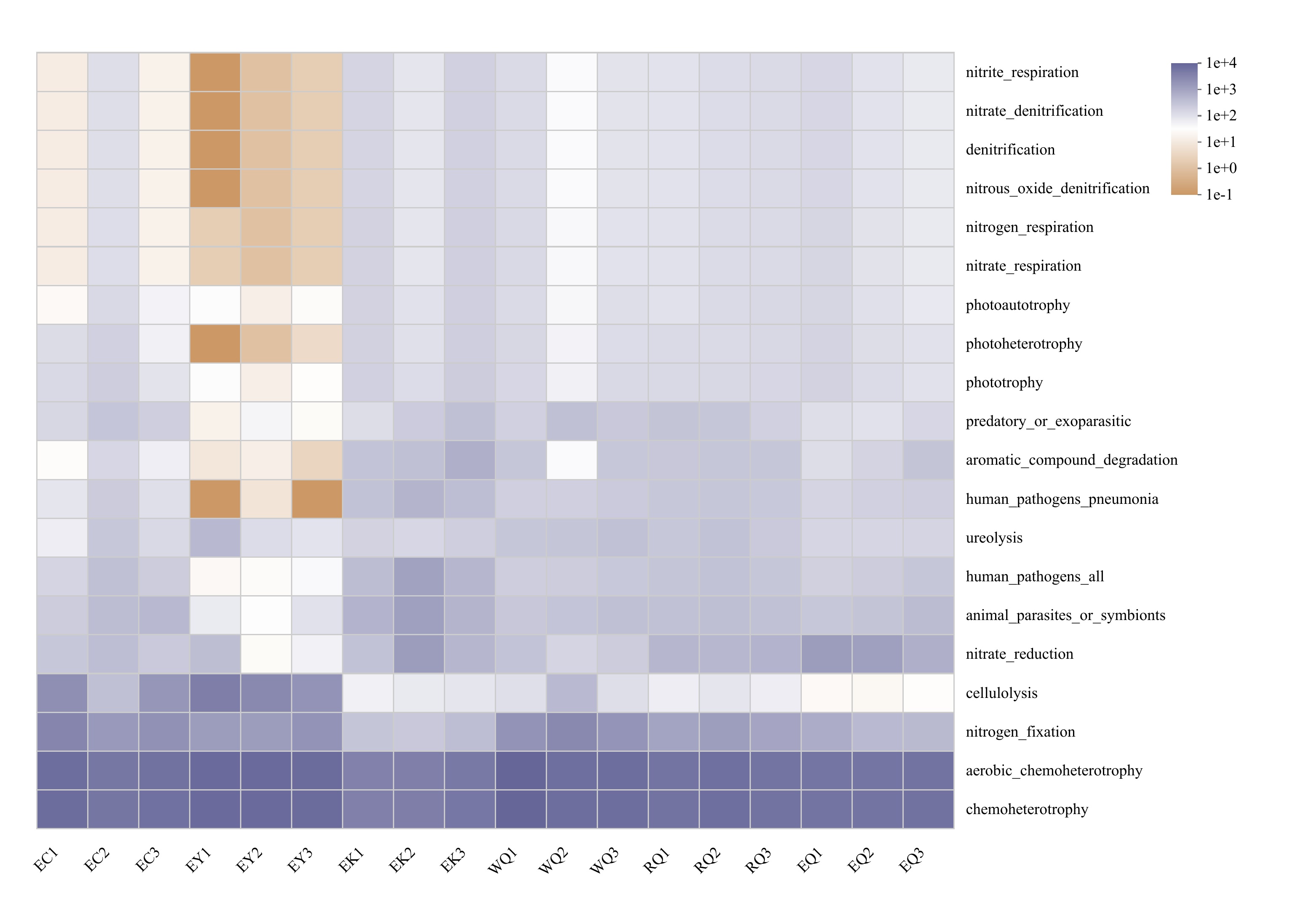

Supplement: Supplementary file 1 [file microorganisms-12-00638-s001.zip › Supplementary Figure S4.tiff]

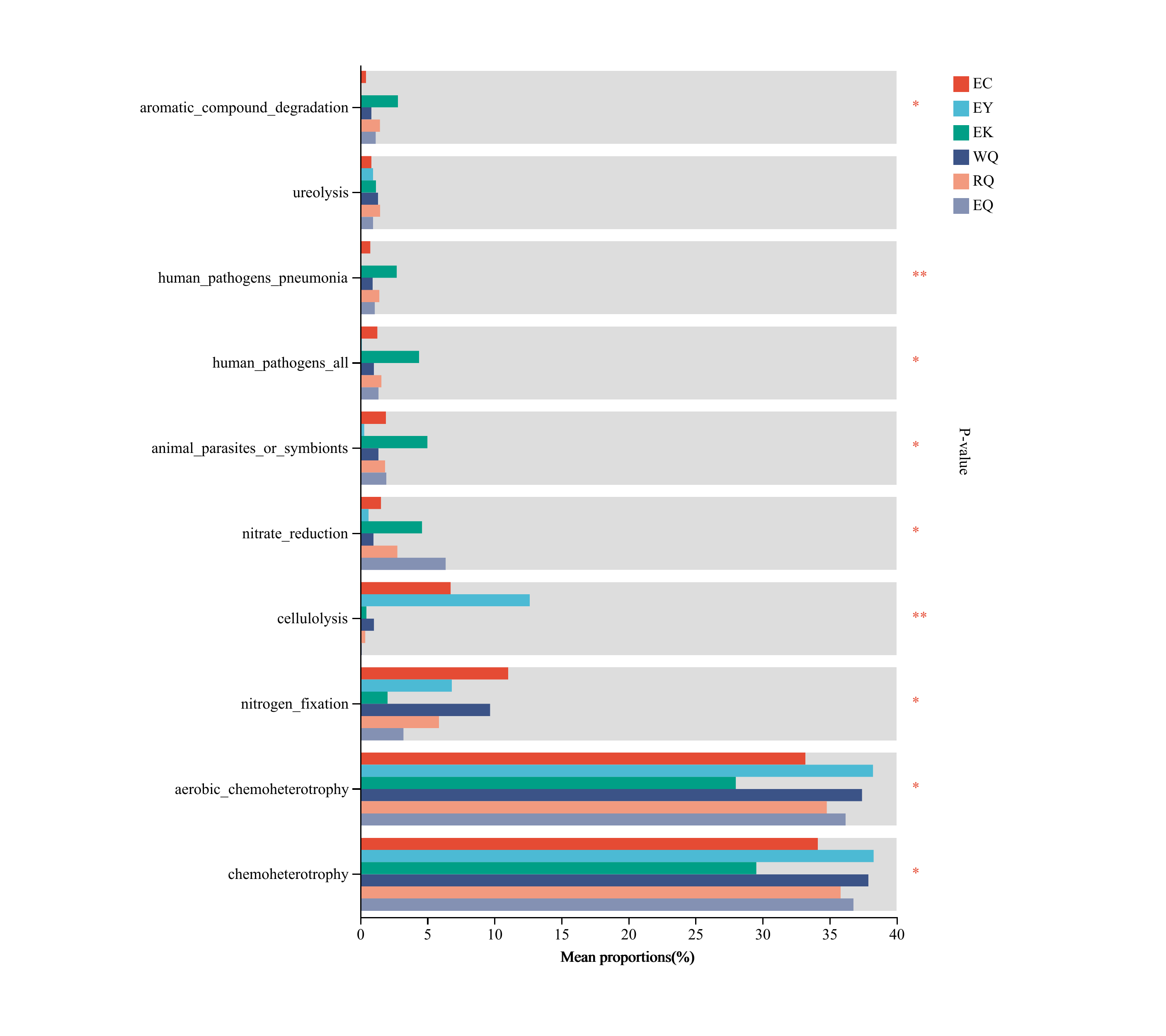

Supplement: Supplementary file 1 [file microorganisms-12-00638-s001.zip › Supplementary Figure S5.tiff]
